# Indirect Electrochemical Oxidation Using Active Chlorine for Treating Distillery Effluent

**DOI:** 10.1002/open.70204

**Published:** 2026-05-08

**Authors:** Perumal Asaithambi, N. M. Hariharan, Madhappan Santhamoorthy, Subramaniapillai Niju, Harshiny M., Arun Thirumurugan, Rajendran Govindarajan

**Affiliations:** ^1^ Department of Biotechnology and Chemical Engineering School of Engineering Faculty of Science, Technology and Architecture Manipal University Jaipur Jaipur Rajasthan India; ^2^ Faculty of Civil and Environmental Engineering Jimma Institute of Technology Jimma University Jimma Ethiopia; ^3^ Department of Biotechnology Vel Tech Rangarajan Dr. Sagunthala R & D Institute of Science and Technology Chennai Tamil Nadu India; ^4^ School of Chemical Engineering Yeungnam University Gyeongsan Republic of Korea; ^5^ Department of Biotechnology PSG College of Technology Coimbatore Tamil Nadu India; ^6^ Department of Biotechnology Easwari Engineering College Chennai Chennai Tamil Nadu India; ^7^ Sede Vallenar Universidad de Atacama Vallenar Chile; ^8^ Department of Biotechnology Hindustan Institute of Technology and Science Chennai Tamil Nadu India

**Keywords:** active chlorine oxidation, electrochemical process, energy consumption, pollutant degradation, stainless steel, titanium with mixed metal oxide, wastewater treatment

## Abstract

All over the world, individuals are worried about the availability of safe drinking water and sanitation. According to the United Nations, there are billions of people who do not have access to clean drinking water. If not adequately treated, wastewater from industrial, domestic, and agricultural operations can harm water quality, human health, and aquatic ecosystems. Electrochemical wastewater treatment technologies are effective, selective, and disinfect in‐situ because of their electrochemical nature. This study aimed to assess the usability and effectiveness of indirect‐electrochemical oxidation (IEO) methods for distillery industrial wastewater (DIW) by measuring treatment efficiency and consumption of electrical energy (CEE). The impact of operational parameters, such as current (0.07–3.4 Amp), pH (3–11), chemical oxygen demand (COD) (500–2500 mg L^−1^), supporting electrolyte concentration (SEC) (2–10 g L^−1^), types of electrolyte (NaCl, KCl, Na_2_SO_4_, and Na_2_CO_3_), and electrode gap (2–4 cm), on the removal of % COD and CEE were investigated. The most effective electrolyte was found to be NaCl. The experiments with COD = 1000 mg L^−1^, SEC = 6 g L^−1^, stirring speed (SS) = 300 rpm, electrode gap (EG) = 2 cm, current = 0.27 Amp, and pH = 6 were established as the optimum level. For these conditions, it was observed that the COD removal was 85% and CEE was 19.38 kWhr kg COD^−1^, respectively. After a period of 6 h of operation, it has been observed that the IEO process offers a significant removal efficiency with respect to the parameters of operation for wastewater. The UV/Vis‐spectrophotometer was employed to evaluate the color removal and oxidation of organic compounds. As a result of the experimental results, the IEO process appears to be a better technology for eliminating contaminants from wastewater while using required electrical energy.

## Graphical abstract

Indirect electrochemical oxidation (IEO) using MMO anode and SS cathode generates active chlorine species that rapidly decolorize and degrade organic pollutants in distillery wastewater. Optimization of current (0.27 A), pH (6), NaCl (6 g L^−1^), and electrode gap (2 cm) resulted in 85% COD removal with low energy demand (19.38 kWh kg^−1^ COD). IEO outperformed with direct EO in both pollutant removal and energy efficiency, supported by UV–Vis evidence of chromophore breakdown.

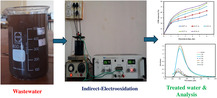

## 1 Introduction

Wastewater from residential, business, and agricultural operations contains a variety of contaminants, such as chemicals, heavy metals, organic waste, and pathogens [1, 2, 3, 4]. These contaminants could be harmful to the ecosystem as well as human health [5, 6]. Untreated wastewater that is released into bodies of water in an unhygienic way contaminates water, disturbs aquatic life, and spreads waterborne diseases [7]. One of the UN Sustainable Development Goals (SDGs), particularly SDG 6 (clean water and sanitation), must be addressed in order to maintain public health, protect aquatic habitats, and facilitate water recycling in regions with water scarcity [8, 9, 10].

The capacity to handle complex pollutants, high energy requirements, and the need for large‐scale infrastructure typically hinder traditional wastewater treatment methods like biological, chemical coagulation, and filtration processes [11, 12, 13, 14]. This has led to an increased need for alternative technologies that are cost‐effective, environmentally benign, and have minimal energy requirements. Because they are environmentally beneficial and can remove a wide range of contaminants, including heavy metals, organic compounds, viruses, microplastics, and nutrients, technologies utilizing electrochemistry have garnered a strong interest in among these possibilities [10, 15, 16]. By driving chemical reactions with electrical currents that break down pollutants and recover valuable resources, electrochemical methods for wastewater treatment provide a novel approach to maintaining water quality [16, 17, 18]. These procedures usually make use of electrodes and electrical energy to trigger chemical reactions that either directly break down pollutants or make it easier to remove them through mechanisms like oxidation, reduction, precipitation, or others. Beginning with the use of electrocoagulation for water purification in the early 20^th^ century, electrochemical wastewater treatment has its origins [4, 19]. Numerous electrochemical methods [20] have been used to the treatment of wastewater, like electro‐oxidation [21], electrocoagulation [22, 23], electroflotation [24], electrocatalysis [25], and electrochemical membrane processes [26, 27], which have evolved over time as a result of advancements in reactor designs, control techniques, and electrode materials. The technologies have now become practical and effective wastewater treatment techniques, offering workable answers to the increasing problems brought on by urban and industrial effluents. Additionally, compared to traditional treatment methods, it offers benefits like exact regulation of treatment procedures, lesser chemical usage and operating costs, and ease of integration through preexisting systems for treating wastewater, making it a desirable option for both municipal and industrial applications [28]. Additionally, by providing eco‐friendly substitutes for traditional chemical treatment techniques, electrochemical treatment methods are consistent with green chemistry concepts. For example, electrochemical oxidation eliminates the need for dangerous substances like ozone (O_3_) or chlorine (Cl_2_) gas by using electricity to produce oxidizing agents like –OH or chlorine species for pollutant degradation [29]. Similar to this, coagulation and flocculation can be accomplished using electrochemical methods like electroflocculation and electrocoagulation without the need for chemical coagulants, which minimizes the use of chemicals and waste generation [30]. Electrochemical treatment improves human health and environmental sustainability by encouraging green chemistry practices [31, 32].

The application of electrochemical technology for wastewater treatment in many sectors and environments has been the subject of numerous studies. Process optimization, reactor designs, electrode materials, operating parameters, and treatment efficiencies are only a few of the topics that have been examined in this research [33, 34, 35]. The efficacy of electrochemical techniques in removing substantial pollutants and generating treated effluents that satisfy legal requirements has been shown by the results. But there are still issues with price, scalability, while developing reliable systems that work in practical conditions.

By evaluating different types of synthetic wastewater, Martínez‐Huitle et al. showed that electrochemical technologies could effectively remove organic matter from actual effluents [36]. The study underlined how crucial it is to generate the right environment in order to completely eradicate organic contaminants. Hand and Cusick investigated electrochemical disinfection in water and wastewater treatment, evaluating its effectiveness in managing both distributed and centralized effluent [37]. The study emphasized how operational considerations and water quality affect how well electrochemical disinfection procedures work. Yang and Qin illustrated the efficacy of electrochemical methods in the retrieval of valuable resources from wastewater by examining the application of cation exchange membranes in electrochemical systems for ammonia recovery [38]. Gao et al. emphasized that electrochemical methods can simultaneously recover resources from wastewater and eliminate persistent contaminants [39]. The study highlighted the advantages of electrochemical treatment techniques in terms of cost‐effectiveness and water saving. Together, these investigations demonstrate the adaptability and efficiency of electrochemical methods in treating a range of wastewater types, including medical and industrial effluents. The study demonstrates the potential of electrochemical methods for resource recovery, sustainable wastewater management techniques, and the removal of impurities.

Even with the advancements in electrochemical wastewater treatment, there are still a number of research gaps that need to be filled. The thorough comprehension of electrochemical processes and the problems they present, including high energy consumption, operational complexity, and environmental danger, is one significant knowledge gap [40]. Mitigation techniques are also required for improved performance and dependability. Closing these gaps will help develop sustainable and efficient wastewater treatment options in addition to advancing basic knowledge of electrochemical methods for wastewater purification. With a focus on their principles, benefits, obstacles, and ways to deal with them, this research study offers an overview of electrochemical methods for wastewater treatment in an effort to enhance treatment methods and solve the increasing challenges of wastewater management. This will enable the provision of clean water for human use.

### 1.1 Mechanisms of Electrochemical Wastewater Treatment

The concepts of electrochemistry are used in electrochemical wastewater treatment methods to produce chemical reactions which alter or eliminate impurities in water [39, 41]. Optimizing treatment efficiency, developing efficient systems, and developing innovative electrochemical technologies all depend on an understanding of the underlying mechanics. Membrane‐assisted methods, electro‐oxidation, electrocoagulation, electroflotation, and electrochemical reduction are all important for treating different forms of wastewater.

Electrochemical wastewater treatment employs electricity to start oxidation, reduction, or electrocoagulation reactions that remove pollutants in water. These reactions happen at the electrodes of an electrochemical cell, where the transfer of electrons causes chemical changes. The underlying process is electro‐oxidation, which uses an external electric potential to oxidize both organic and inorganic molecules at the anode [8, 42]. During the process, extremely reactive oxidizing species are generated, such as hydroxyl radicals (OH) and chloride species (Cl_2_/ClO) [8]. These species attack and break down organic compounds into less hazardous and simpler byproducts. Electro‐oxidation has various benefits, such as high productivity, selectivity, and the ability to turn organic contaminants into CO_2_, H_2_O, and inorganic ions [40, 43]. It works especially well for resistant substances like industrial chemicals, insecticides, and medications [44, 45]. A compilation of important electrochemical treatment studies that include a variety of wastewater types is presented in the comparative Table 1. The table also highlights the electrode materials and operating parameters that were utilized. These studies provide a summary of the removal efficiencies and energy consumption that have been documented in the relevant existing research work. By highlighting the technological variances and performance gaps that are addressed in this study, this review assists in positioning the current work within the context of current research.

**TABLE 1:** Electrochemical oxidation studies for various wastewater.

Types of wastewaters	Electrode type	Operating conditions	Removal efficiency, %	Energy consumption	Ref.
Sulfur dyes	Mixed metal oxide (MMO) plate	Treatment time: 40 min, sulfur dye: 100 mg/L, NaCl: 6000 mg/L, voltage: 12 V, current: 5 A, and temperature: 27°C–32°C	Sulfur dyes: 100	13.3 kWh/m^3^	[46]
Fabric dyeing	Graphite	pH: 6, current density: 50 mA/cm^2^; time: 80 min.	Color: 99 COD: 80	—	[47]
Park wastewater	Mesh‐plate Ti/PbO_2_: Anode Ti: Cathode	Volume: 2.8 m^3^, surface‐to‐volume ratio: 17.14 m^2^/m^3^, current density: 100 A/ m^2^, flow velocity: 14.0 m/h.	COD: 60 Color: 84	43.5 kWh/kg COD	[44]
Restaurant	Multi carbon	Chloride ion: 2 g/L, pH: 5, distance between electrode: 10 cm, voltage: 12 V, time: 90 min	COD: 92.84	‐‐	[48]
Per‐and poly‐fluoroalkyl substances (PFAS).	Boron‐doped diamond (BDD)	Current density: 14 mA cm^−2^, treatment: 3–4 h, Na_2_SO_4_: 14.2 g/L	PFAS:99.50	‐‐	[49]
Cooking	Ti/RuO_2_–IrO_2_	Electrode gap: 0.5 cm, current density: 15.6 mA/cm^2^, treatment: 60 min, COD: 178.0–285.0 mg/L, NH_4_^+^‐N: 55.0–76.0 mg/L	COD: 62 NH_4_^+^‐N: 96	8.60 kWh/m^3^	[50]
Oil wastewater	Ti/Iro_2_	Current density: 16.67 mA/cm^2^, concentration of sulfide: 50 mg/L, time: 25 min	Sulfide:100	—	[51]
Petroleum	Graphite and stainless‐steel electrodes	Current density of 3 mA/cm^2^ and time 15 min, initial concentration of about 6.8 mg/L	In terms COD & BOD: 50%–60%	0.79 kWh/m^3^ and operating cost was 0.051 $/m^3^	[52]
Synthetic dye		Electrolyte concentration: 0.5 g/L, current density: 1.25 A/dm^2^, pH: 3, electrode distance: 1 cm	Decolorization: 94.79 COD: 76.82	—	[53]
Heavy metals	TiO_2_‐NTs/GO/SnO_2_	pH: 3–9, current: 0.1–0.9 A, time: 30–180 min,	Cu: 97.1 Zn: 95.7 COD: 91.2	7.32 kWh/m^3^	[54]
Disinfecting wastewater	MMO anode	Current density: 7.14 mA/cm^2^, NaCl:0.2 g/L, treatment time: 9 min, flow rate: 40 mL/min.	Bacterial inactivation: 96	0.184 kWh/m^3^	[55]
Wastewater	Graphite‐ anode and SS ‐ cathode	Contact time: 5 min, TDS:2000 mg/L, CD: 4 mA/cm^2^	Complete removal *of E. coli*	0.5 kWh/m^3^	[56]
Synthetic bilge water	Pt/Ti mesh: anode pure mesh Ti as cathode	Current density: 10 mA/cm^2^, pH: 7.5, and flow rate: 20 mL/min	COD: 80 Oil and grease: 99	—	[57]

Prior research has shown the benefits of using an indirect electrochemical technique to reduce pollutants from synthetic wastewater. On the other hand, there have been a very limited number of studies conducted with real industrial wastewater systems. Electrical energy consumption is a crucial component of both direct and indirect electrochemical processes, in addition to eliminating pollutants from wastewater and industrial effluent from a functional and financial perspective. This study was undertaken to comprehensively assess both direct and indirect electrochemical processes for removing color and COD from distillery wastewater, along with estimating the associated electrical energy consumption. The effect of operating parameters such as current and electrolysis time, solution initial pH, initial COD concentration, electrode gap, electrolyte concentration, and electrolyte type was also examined.

## 2 Material and Methods

### 2.1 Wastewater Characteristics

The wastewater was acquired from a distillery industry which was located in the India, state of Tamilnadu. Numerous factors, such as total dissolved solids (TDS), biological oxygen demand (BOD), chemical oxygen demand (COD), pH, and total suspended solids (TSS), were analyzed through the examination of the effluent. Among the key parameters of the wastewater are the following: COD: 80,000–90,000 mg L^−1^, pH: 4.1–4.3, TSS: 15.44 g L^−1^, BOD: 7,000–8,000 mg L^−1^, and TDS: 5,550–5,750 mg L^−1^, as wells as dark brown in color, with a smell of charred sugar.

### 2.2 Experimental Techniques

**FIGURE 1:**
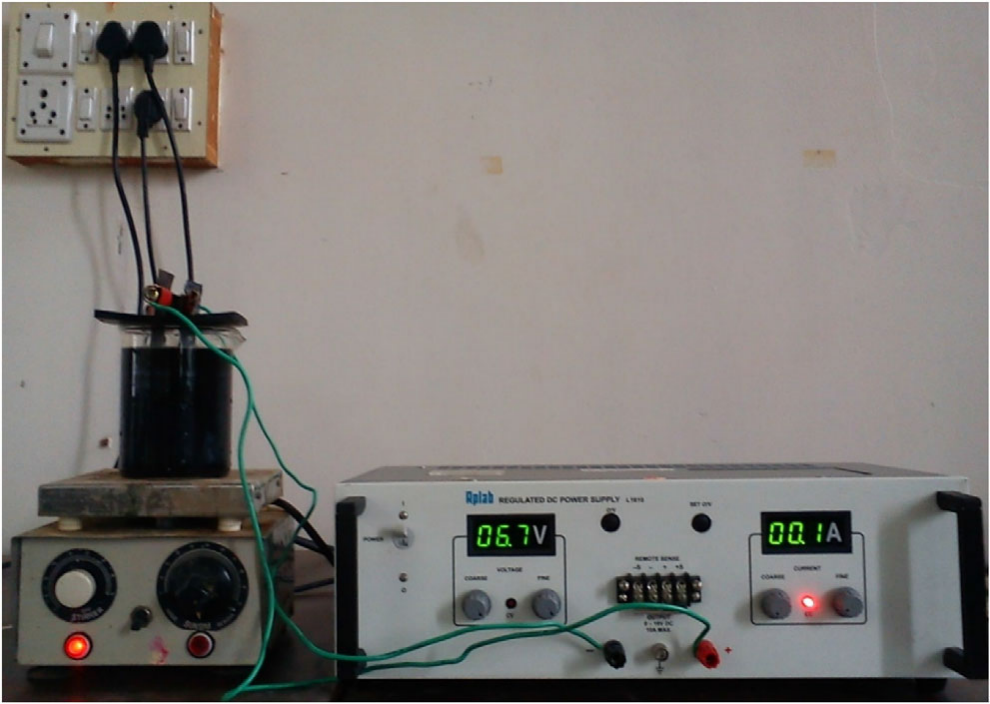
Indirect electrochemical oxidation treatment cell.

Each experiment employed an electrochemical reactor with a net capacity of 600 mL, constructed of Perspex glass. Figure 1 shows a schematic representation of the lab‐scale batch experimental setup. An undivided cell with two electrodes and a direct‐current (DC) rectifier made up the electrochemical oxidation apparatus. Titanium mesh coated with mixed metal oxide (MMO) and stainless steel (SS) electrodes, provided by M/s. Titan Anode Fabricators Private Limited, Chennai, Tamilandu, India, was utilized as the anode and cathode, respectively. The anode and cathode have the dimensions of 7.25 cm × 6.0 cm × 0.1 cm and were placed vertically and parallel to each other with an interelectrode distance of 2 cm. The void fraction of the mesh type anode accounts 20% by area. Hence, the available effective electrode area is 34.8 cm^2^ for anodic reactions. The electrode plates were cleaned manually by washing in distilled water prior to every run. An electrical DC power source (APLAB Ltd; Model L1606) was linked to the electrodes of the electrochemical reactor, which had a 500‐mL effluent volume. The content of COD in the wastewater was between 500 and 2500 mg L^−1^. The untreated industrial effluent functioned as the dilution factor for solution preparation. The required current was provided to both the anode and the cathode while they were submerged in the effluent. An electric magnetic stirrer (MS7H550 Pro) was utilized in order to maintain a consistent speed while stirring the solution. All experiments were conducted at a controlled temperature of 35°C ± 1°C. Through the utilization of a pH meter (Elico; Model LI120), the pH of the solution was determined. In the course of the research, the initial pH was modified by employing a reagent consisting of either 0.1 N NaOH or 0.1 N H_2_SO_4_. Before the samples were analyzed for color and COD elimination, they were taken from the reactor at predetermined intervals of time and centrifuged (REMI, Model: R‐24) for 15 min at a speed of 15 000 rpm. All electrochemical experiments were conducted in triplicate, and the corresponding mean values are presented.

### 2.3 Analysis

COD was used to quantify the sample's pollutant concentration. The dichromate closed reflux method was utilized all throughout the process of determining the COD value. With a UV/Vis‐Spectrophotometer [Jasco, V‐570], the colors of the untreated and treated samples were analyzed at a maximum wavelength of 300 nm.

### 2.4 COD and Color Removal (%)

The COD and color removal % of the effluent were calculated using Equations (1) and (2).

(1) $$\text{COD removal},\left(\%\right)=\left(\frac{COD_{0}-COD_{t}}{COD_{0}}\right)*100$$

The COD at time *t* = 0 (initial) and at *t* (reaction time) is represented by COD_0_ and COD_*t*_ (in mg L^−1^), respectively.

(2) $$\text{Color removal},\left(\%\right)=\left(\frac{Abs_{0}-Abs_{t}}{Abs_{0}}\right)*100$$

The absorbance at time *t* = 0 (initial) and at time *t* (reaction time) are denoted by *Abs*_0_ and *Abs*_*t*_, respectively.

### 2.5 Consumption of Electrical Energy (CEE)

The amount of electrical energy used to remove 1 kg of COD from the effluent is known as consumption of electrical energy (kWh kg^−1^), and it can be computed using Equation (3).

(3) $$\text{Consumption of electrical energy}\frac{VIt}{3600*10^{3}}*\frac{1}{\left(COD_{0}-COD_{t}\right)*V_{R}*10^{-6}}$$

where *V* is the observed cell voltage (V), *I* is the current (A), and *V*_*R*_ is the effluent volume (L).

### 2.6 Current Efficiency

Using the COD value and Equation (4), the current efficiency for the anodic oxidation of the organic compounds in the effluent was calculated.

(4) $$\text{Current efficiency}=\frac{\left({\mathrm{COD}}_{0}-{\mathrm{COD}}_{t}\right)FV_{R}}{8I\Delta t}*100$$

*F* is the Faraday constant (96487 C mol^−1^), and 8 is the oxygen equivalent mass (g equiv^−1^).

## 3 Results and Discussion

### 3.1 Process Optimization for Electrochemical Oxidation Process

For the purpose of enhancing the efficiency of the indirect electrochemical oxidation, the following aspects were examined and addressed in more depth: a solution with a pH ranging from 2 to 10, a chemical oxygen demand (COD) extending from 500 to 2500 mg L^−1^, a concentration of electrolyte (NaCl) ranging from 2 to 10 g L^−1^, and an electrode gap from 2 to 4 cm.

#### 3.1.1 Effects of Current and Electrolysis Time

**FIGURE 2:**
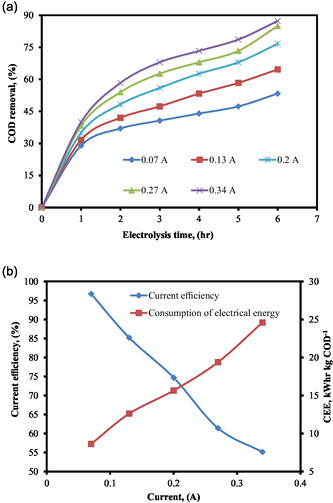
(a) % COD removal at various current as a function of electrolysis time. (b) Influence of current on current efficiency and CEE (pH = 6, COD = 1000 mg L^−1^, supporting electrolyte concentration (SEC) = 6 g L^−1^, stirring speed (SS) = 300 rpm, electrode gap (EG) = 2 cm).

In the electro‐oxidation process, which is used for the treatment of wastewater, the applied current and the electrolysis time are highly important functions [8, 58, 59]. Both the cost and the amount of energy that is consumed by the electrolysis process are directly determined by these parameters [32, 58]. The effect of current as a function of treatment time on the electro‐oxidation was carried out at the COD = 1000 mg L^−1^, SEC = 6 g L^−1^, SS = 300 rpm, EG = 2 cm, and pH = 6 with different current 0.07, 0.13, 0.20, 0.27, and 0.34 Amp. It is seen in Figure 2a that the COD removal effectiveness varies depending on the current. In accordance with the findings presented in Figure 2a, it was demonstrated that the % of COD that was removed increased in proportion to the rise in the current.

In order to investigate the effects of current on electrical energy consumption and current efficiency in the treatment of the distillery effluent at an initial COD concentration of 1000 mg L^−1^, NaCl was employed as a supporting electrolyte at a concentration of 6 g L^−1^ in the solution. The results are presented in Figure 2b. The figure illustrates that electrical energy consumption increased while current efficiency decreased as the current increased. At currents exceeding 0.27 Amp, the percentage variation in COD removal was minimal. An additional increase in the process current may lead to higher operating costs owing to increased electrical energy consumption resulting from decreased current efficiency. This happens because electro‐generated active chlorine facilitates the oxidation of the organic component within the bulk solution rather than immediately on the electrode surface. At larger currents, the performance of the process is only marginally more effective [32]. Additionally, it was noted that an increase in current also leads to more undesirable reactions and the loss of electrical energy in the form of heat [8]. As a result, for future experiments, the current was set to 0.27 Amp.

#### 3.1.2 Impact on pH

**FIGURE 3:**
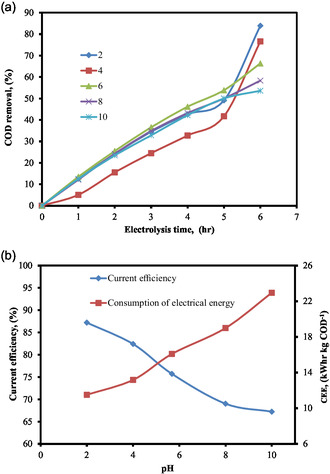
(a) % COD removal by electrolysis time at various pH levels. (b) Effect of pH on current efficiency and CEE (COD = 1000 mg L^−1^, SEC = 6 g L^−1^, SS = 300 rpm, EG = 2 cm, and current = 0.27 Amp).

In an electrochemical process, the pH of the solution has a significant impact on the efficiency with which pollutants are removed from wastewater [46, 60]. To study this relationship, electrolysis tests were conducted throughout a pH range of 2–10, delivering a constant current of 0.27 Amp and altering the period from 1 to 6 h. As seen in Figure 3a, raising pH from 2 to 10 leads to a progressive drop in COD removal efficiency rates. Two important equilibria, Cl_2_/HClO and HClO/ClO^−^, control the production of oxidizing agents and influence the distribution of stable active chlorine species. While ClO^−^ is preferred in alkaline settings, HClO and Cl_2_ predominate in acidic (low pH) situations. Wastewater degradation occurs more quickly at pH 3 than in basic conditions because HClO and Cl_2_ have larger redox potentials (1.49 V/SHE and 1.36 V/SHE, respectively) than ClO^−^ (0.89 V/SHE) [48]. Wastewater molecules are protonated at acidic pH values, whereas chlorine often occurs as hydrated Cl_2_, which interacts more easily. HClO is the most common oxidant between pH 3 and 7. Its pollutant elimination is slower than at lower pH levels, even though active chlorine production peaks at pH 5, indicating that Cl_2_ is the most efficient oxidant of the three chlorine species. ClO^−^ predominates at pH > 8; however, degradation rates are lower due to its lesser oxidizing potential [46].

In the treatment of distillery effluent with an initial concentration of 1000 mg L^−1^ of COD, sodium chloride (NaCl) was used as the supporting electrolyte, and a solution concentration of 6 g L^−1^ was used. The purpose of this study was to investigate the impact of pH on current efficiency and electrical energy consumption. As can be seen in Figure 3b, an increase in the pH of the solutions resulted in a decrease in the current efficiency as well as an increase in the quantity of electrical energy that was consumed.

#### 3.1.3 Influence COD Concentrations

**FIGURE 4:**
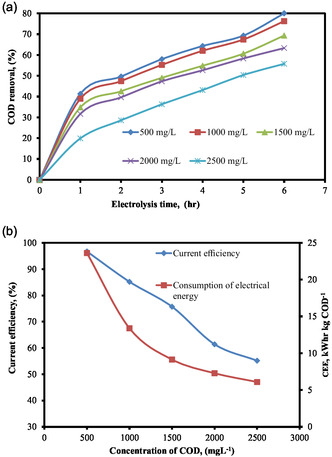
(a) % COD removal by electrolysis time for varying starting COD concentrations. (b) Effect of initial COD concentration on current efficiency and CEE (pH = 6, SEC = 6 g L^−1^, SS = 300 rpm, EG = 2 cm, and current = 0.27 Amp).

It is necessary for the EO process to take into consideration the type of water and starting concentration of effluent [46]. Different organic and mineral substance loading in water supplies often impacts the % of pollutants removed [61]. The majority of experimental research has been on ultrapure and pure water, although these impractical systems are entirely different from actual circumstances. The effect of COD concentrations as a function of time on the electro‐oxidation was carried out at the pH = 6, SEC = 6 g L^−1^, SS = 300 rpm, EG = 2 cm, and current = 0.27 Amp with different CODs 500, 1000, 1500, 2000, and 2500 mg L^−1^. This is demonstrated in Figure 4a, which depicts the COD removal efficiency at various COD levels. The figure indicates that the proportion of COD removed dropped as COD concentrations increased.

A current of 0.27 Amp was used to investigate the effects of varying COD concentrations on the efficiency of the current and the amount of electrical energy that was consumed. The initial concentration of COD was 1000 mg L^−1^. In accordance with what is depicted in Figure 4b, the current efficiency and the amount of electrical energy that is consumed both decrease as the concentrations of COD increase. The restricted oxidizing species that are readily available namely ClOH^−^, Cl_2_^−^, and ClO_3_^−^ at a given electrolyte concentration of 6 g L^−1^ are the cause of this decline in COD elimination, current efficiency, and electrical energy consumption [62]. This occurs concurrently with a rise in the initial COD concentration.

#### 3.1.4 Effects of Electrolyte Concentrations

The effect of supporting electrolyte concentration of NaCl on the % pollutant removal has been reported elsewhere that NaCl is very effective in the destruction of organics present in the effluent and decolorization can be completely achieved [63, 64]. The addition of the supporting electrolyte which allows the removal efficiency increases and a degradation of pollutants occurs due to the participation of active chlorine through the possible “direct” and “indirect” roles for the chloride anion in the electrochemical reaction. In indirect electro‐oxidation, NaCl is added to increase the conductivity and generates hypochlorite ions. The anodic reaction is given as:

(5) $$2Cl^{-}\to Cl_{2(g)}+2e^{-}$$

(6) $${\mathrm{Cl}}_{2(g)}+H_{2}O\to H^{+}+{\mathrm{Cl}}^{-}+\mathrm{HOCl}$$

(7) $$\mathrm{HOCl}↔H^{+}+{\mathrm{OCl}}^{-}$$

(8) $$\text{Pollutants}+OCl^{-}\to CO_{2}+H_{2}O+Cl^{-}+\text{Products}$$

The generated hypochlorite ions act as the main oxidizing agent in the pollutant degradation. In the indirect electro‐oxidation rate of organic pollutants, a reaction sequence of chloride–chlorine–hypochlorite–chloride takes place in the bulk [65].

**FIGURE 5:**
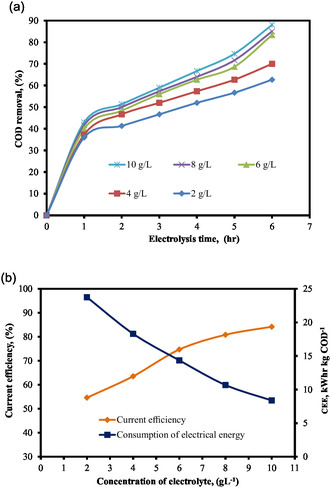
(a) % COD removal at various supporting electrolyte concentrations as a function of electrolysis time. (b) Effect of supporting electrolyte concentrations on current efficiency and CEE (pH = 6, COD = 1000 mg L^−1^, SS = 300 rpm, EG = 2 cm, and current = 0.27 Amp).

Figure 5a illustrates how the concentration of electrolytes affects the elimination of COD by the EO process. The electrochemical results in Figure 5a reveal that increasing NaCl concentrations improves COD removal efficiency and treatment time. A maximum COD removal percentage of 85% was achieved with an electrolyte content of 10 g L^−1^ for an initial COD concentration of 1000 mg L^−1^. The increase in COD removal rate could be attributed to the production of oxidizing species such as ClOH^−^, Cl_2_, and ClO_3_^−^ near the surface as a result of chloride oxidation [66]. The generation of oxidizing species close to the surface through chloride oxidation may be the primary cause of the increase in COD removal rate. In further research, optimum NaCl content was 6 g L^−1^ because raising the level to 10 g L^−1^ did not yield any discernible benefits.

The current efficiency and consumption of electrical energy were investigated with an initial COD of 1000 g L^−1^ and a current of 0.27 Amp. Figure 5b demonstrates that when the concentration of electrolyte increases, current efficiency increases while electrical energy consumption reduces. The current efficiency also increased dramatically as chloride ion concentrations increased. When NaCl is present in the reaction medium, it produces highly strong oxidants of HOCl/ClO^−^ in situ, which raise the electrolyte concentration. The voltage then immediately lowers the concentration of these chemicals in the medium, allowing for a quicker elimination of COD. Anode and cathode potentials, as well as cell voltage, may vary as a result of the reactions’ kinetic properties.

#### 3.1.5 Effect of Supporting Electrolyte Types

NaCl is an electrolyte with numerous benefits over commonly employed salts as KCl, Na_2_SO_4_, and Na_2_CO_3_ in the EO treatment of wastewater [17, 58, 67]. NaCl has two key functions throughout the EO process: (i) increasing conductivity, therefore reducing electricity use, and (ii) producing hypochlorite at the anode and subsequently taking part in the solution's organic materials’ indirect oxidation processes. However, adding too much NaCl salt will result in the production of carcinogenic byproducts called chlorinated organics. Cl_2_ and ClO^−^ can do the indirect oxidation in the solution. Chlorine ions (such as Cl_2_ and ClO^−^) can considerably lessen the inhibitory effects of other ions, such as HCO_3_^−^ and SO_4_^2−^, during the EO treatment in addition to contributing ions in the charge transport process. Ca^2+^ and Mg^2+^ ions will precipitate as a result of these ions, covering the electrode surface with an insulating layer [58].

**FIGURE 6:**
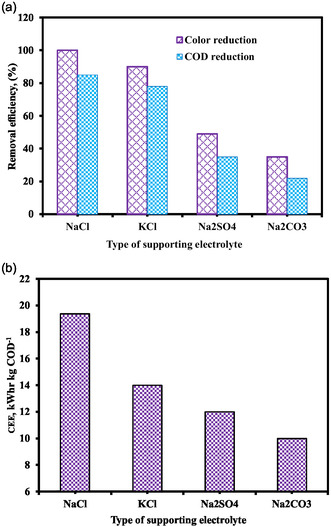
Effect of types supporting electrolyte on (a) color and COD reduction, (%) (b) CEE (pH = 6, COD = 1000 mg L^−1^, SEC = 6 g L^−1^, SS = 300 rpm, EG = 2 cm, and current = 0.27 Amp).

The effects of the supporting electrolyte type were assessed using NaCl, KCl, Na_2_SO_4_, and NaNO_3_. The color and COD reduction of distillery wastewater were used to assess the impact of various electrolytes. The color and COD removal efficiency varied according to the type of supporting electrolyte, as Figure 6a illustrates. Following 6 h of electro‐oxidation, the color and COD removal efficiency for NaCl, KCl, Na_2_SO_4_, and NaNO_3_ were 100%, 90%, 49%, and 35% and 85%, 78%, 35%, and 22%, respectively. Because NaCl produced the greatest color and COD elimination, it was selected as the supporting electrolyte for the ensuing studies. On the anode surface, Cl^−^ ions from NaCl are transformed into Cl_2_, producing active chlorine species including ^•^Cl, HOCl, and ClO^−^ that improve removal efficiency through indirect oxidation [17]. KCl as the supporting electrolyte produced the second‐highest color and COD removal effectiveness after NaCl. The Na_2_SO_4_ as the supporting electrolyte produced the third‐highest color and COD removal efficiency. This is due to the formation of S_2_O_8_^2−^ and sulfate radicals, which indirectly oxidize wastewater, when Na_2_SO_4_ is utilized as the supporting electrolyte. Nevertheless, the removal effectiveness is less than that of NaCl because SO_4_^2−^ also lowers the quantity of OH. Na_2_NO_3_ was found to have the lowest color and COD removal effectiveness when utilized as the supporting electrolyte due to its absence of ions that would cause indirect oxidation.

As can be seen in Figure 6b, the amount of electrical energy that was consumed changed based on the type of electrolyte that was supporting the system. The concentrations of electrical energy that were consumed by NaCl, KCl, Na_2_SO_4_, and Na_2_NO_3_ after 6 h of electro‐oxidation were 19.38, 14, 12, and 10 kWh kg^−1^ COD, respectively. As a result, NaCl was selected as the supporting electrolyte for the upcoming experiments since it produced the maximum COD removal with the necessary electrical energy consumption.

#### 3.1.6 Effect of Electrode Gap

**FIGURE 7:**
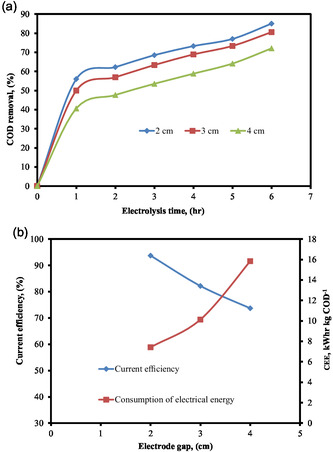
(a) % COD removal as a function of electrolysis time at various electrode gaps. (b) Effect of different electrode gap on current efficiency and CEE (pH = 6, COD = 1000 mg L^−1^, SEC = 6 g L^−1^, SS = 300 rpm, and current = 0.27 Amp).

The distance between electrodes in an electrochemical system has a significant impact on electrochemical capacity. As the distance between two electrodes increases, the solution layer between them becomes more resistant, there is less transit of impurities, and the ability to diffuse ions to the electrode's working surface decreases. If the distance between the two electrodes is too small, on the other hand, there will be uneven electric field distribution or ion density concentrated in a narrow zone that is too large [68]. These conditions will either prohibit the redox process from taking place on the electrode surface or reduce the effectiveness of pollution remediation at the electrode surface. The experiment was carried out at different inter‐electrode distances to examine the impact of electrode distance on COD elimination. Figure 7a shows how the spacing between electrodes affected the elimination of COD. It is evident from Figure 7a that the distance between electrodes had an impact on the elimination of COD from wastewater using electrochemical oxidation treatment. The COD removal efficiency increased as the distance between the cathode and anode decreased during the electrolysis period of 1–6 h.

Under the following operating conditions: pH = 6, COD = 1000 mg L^−1^, SEC = 6 g L^−1^, SS = 300 rpm, and current = 0.27 Amp, the effect of electrode gap on the EO process was investigated using measurements from 2 to 4 cm. Figure 7b illustrates that augmenting the electrode gap from 2 to 4 cm decreased the current efficiency and elevated the consumption of electrical energy from 7.42 to 15.84 kWhr kg COD^−1^. Ohmic losses associated with anode and cathode overvoltage and mass transfer resistance limit anodic oxidation, which results in a decrease in ions at the anode as the electrode gap widens. On the other hand, the electrolytic process is aided by the reduced resistance of current flow in solution when the electrode is kept to a minimum, increasing the % of COD elimination [69]. In order to decrease electrical energy consumption and increase the effectiveness of COD removal, the optimal interelectrode distance is determined to be 2 cm.

### 3.2 Comparison of Direct/Indirect Electro‐oxidation Process

**FIGURE 8:**
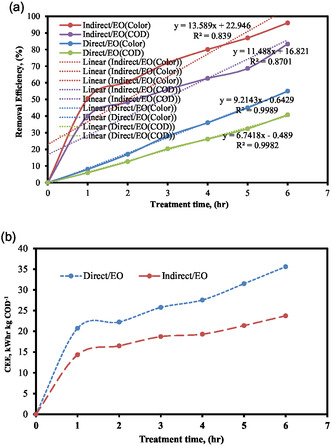
Comparison of direct and indirect electro‐oxidation process on (a) color and COD removal efficiency, and (b) CEE (pH = 6, COD = 1000 mg L^−1^, SEC = 6 g L^−1^, SS = 300 rpm, EG = 2 cm, and current = 0.27 Amp).

The optimal circumstances were utilized in order to evaluate the effectiveness of color and COD removal, as well as the consumption of electrical energy, in both the direct and indirect electro‐oxidation processes. Figure 8a,b presents the findings of the investigation. Figure 8a compares the color and COD removal efficiencies of direct electro‐oxidation (direct‐EO) and indirect electro‐oxidation (IEO) under identical optimized operating conditions (pH = 6, COD = 1000 mg L^−1^, NaCl = 6 g L^−1^, SS = 300 rpm, EG = 2 cm, current = 0.27 Amp). As presented in the Figure 8a, the two processes produce markedly different removal outcomes: after 6 h of treatment, the direct‐EO process achieves 83.33% color removal and 30.75% COD removal, respectively, whereas the IEO process achieves color and COD removals of approximately 96% and 40.77%. This difference arises from the fundamental reaction pathways involved in each process. In direct‐EO, oxidation occurs primarily on the anode surface, where hydroxyl radicals generated at the MMO anode directly degrade organic molecules. In contrast, IEO relies on chlorine‐based oxidants (Cl_2_, HOCl, ClO^−^) formed from chloride ions present in the electrolyte. These species participate in bulk‐phase oxidation and are effective at decolorization but less aggressive toward complete mineralization of organics, resulting in lower COD removal efficiencies. This mechanistic distinction explains why both color and COD removal are consistently higher in the IEO process than in the direct‐EO pathway, as shown in Figure 8a. Furthermore, the studies clarifies that although IEO provides higher pollutant removal, the IEO process is superior in terms of electrical energy consumption, achieving higher removal efficiencies per unit of energy input relative to direct‐EO, as seen in Figure 8. This distinction supports the conclusion that IEO is more energy‐efficient and thus more suitable for large‐scale wastewater applications where operational cost is a critical factor.

Figure 8b illustrates the consumption of electrical energy by two processes, namely the direct electro‐oxidation process and the IEO process, for the removal of color and COD. The findings of the study indicated that the IEO method was superior to the direct‐electro‐oxidation process. In the indirect/EO process, the removal of color and COD from distillery wastewater was higher, and the consumption of electrical energy was lower than in the direct/EO process. This is obvious from the data presented in Figure 8a,b. As a result, when the direct/EO process and the indirect/EO process were compared for the removal of color and COD percentages with the amount of electrical energy consumed by distillery wastewater, the indirect/EO process was found to be more suited than the direct/EO method.

### 3.3 Kinetic Analysis

Electrochemical oxidation processes frequently follow pseudo–1^st^‐order kinetics, where the degradation rate of organic matter is proportional to its instantaneous concentration. Based on COD decay profiles obtained at different treatment process, the degradation of COD in distillery effluent was modeled using the standard pseudo–1^st^ order kinetic expression:

(9) $$ln\left(\frac{{\mathrm{COD}}_{0}}{{\mathrm{COD}}_{t}}\right)=kt$$

where ${\mathrm{COD}}_{0}$ = initial COD concentration, ${\mathrm{COD}}_{t}$ = COD at time t,andk = kinetic rate constant (min^−1^).

Under optimized conditions (pH = 6, current = 0.27 Amp, NaCl = 6 g L^−1^, EG = 2 cm, COD_0_ = 1000 mg L^−1^), the COD decay curve showed excellent linearity when plotted as $\ln({\mathrm{COD}}_{0}/{\mathrm{COD}}_{t})$ versus time, indicating pseudo–1^st^‐order behavior. The calculated rate constant k with different treatment process, which agrees with known electrochemical oxidation behavior. The kinetic trends are consistent with the formation of reactive chlorine species such as Cl_2_, HOCl, and ClO^−^, which dominate the indirect oxidation pathway in chloride‐supported systems.

### 3.4 Instrument Analysis

**FIGURE 9:**
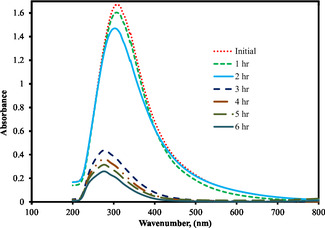
The characteristic of UV/Vis spectra for treatment of distillery effluent by indirect electrochemical oxidation.

The present study primarily focuses on evaluating treatment efficiency and energy consumption; however, the formation and transformation of intermediate species were also examined qualitatively through UV–Vis spectrophotometric analysis. The UV–Vis spectra of the distillery effluent before and after IEOx treatment (Figure 9) exhibit a pronounced reduction in absorbance peaks associated with chromophoric organic constituents. The initial UV–Vis spectrum shows a strong absorption peak around 280–300 nm and a broad absorbance tail extending into the visible region (400–600 nm), which correspond to π–π* transitions of aromatic rings and conjugated structures characteristic of melanoidins and other phenolic compounds commonly present in distillery wastewater. During electrochemical treatment (1–6 h), a substantial decrease in absorbance is observed across the entire spectrum. The progressive reduction of the 300 nm peak indicates the breakdown of aromatic structures, while the disappearance of the broad visible‐region absorbance suggests degradation of high‐molecular‐weight melanoidin polymers into smaller, less conjugated intermediates. The overall hypochromic effect, without significant wavelength shift, confirms that chromophore destruction is the dominant mechanism. By 5–6 h, the marked decline in both UV and visible absorbance demonstrates extensive mineralization or transformation of complex organics, correlating well with the observed decolorization efficiency and validating the effectiveness of the treatment process.

## 4 Conclusion

This study demonstrates that electrochemical oxidation, particularly the indirect EO process, is a highly effective and energy‐efficient method for treating distillery industrial wastewater. Both direct and indirect EO treatments successfully reduced color and COD; however, the indirect EO system consistently exhibited superior performance, achieving higher pollutant removal while requiring lower electrical energy input. The study systematically evaluated the influence of operational parameters—including pH, current, initial COD concentration, supporting electrolyte concentration, electrolyte type, and electrode gap—on pollutant degradation behavior and energy consumption. Results show that neutral pH, lower COD loading, higher NaCl concentration, a 2‐cm electrode gap, and a current of 0.27 A collectively supported the highest removal efficiency and optimal energy use. With the MMO/SS electrode pair, increasing current, NaCl concentration, and treatment duration significantly enhanced color and COD removal, while excessive electrolyte levels or electrode spacing increased energy consumption without added benefit. Findings confirm that the indirect EO process generates in situ oxidants (HOCl/ClO^−^/Cl_2_) that accelerate degradation, making it more effective than direct oxidation for real industrial effluents. The UV–Vis spectral analyses further validated the substantial breakdown of chromophoric organic compounds during EO treatment. Overall, the optimized indirect EO system provides a robust, scalable, and environmentally friendly option for treating high‐strength distillery wastewater. Its reduced chemical demand, enhanced controllability, and lower energy requirements highlight its potential for integration into industrial wastewater treatment facilities. These outcomes reinforce the relevance of EO technologies toward achieving the United Nations Sustainable Development Goal (SDG 6): ensuring access to clean water and effective sanitation through advanced, sustainable wastewater management solutions.

## Author Contributions

**Perumal Asaithambi**: investigation, data curation, resources; writing – original draft. **N.M. Hariharan**: conceptualization, methodology, validation, supervision. **Madhappan Santhamoorthy**: conceptualization, methodology, validation, supervision. **Subramaniapillai Niju**: conceptualization, methodology, validation, supervision. **Harshiny M**: conceptualization, methodology, validation, supervision. **Arun Thirumurugan**: conceptualization, methodology, validation, supervision. **Rajendran Govindarajan**: conceptualization, methodology, validation, supervision.

## Funding

This research did not receive any specific grants from funding agencies in the public, commercial, or not‐for‐profit sectors.

## Conflicts of Interest

The authors declare no conflicts of interest.

## Data Availability

The authors do not have permission to share data.
